# The impact and determinants of the energy paradigm on economic growth in European Union

**DOI:** 10.1371/journal.pone.0173282

**Published:** 2017-03-16

**Authors:** Jean Vasile Andrei, Mihai Mieila, Mirela Panait

**Affiliations:** 1 Business Administration Department, Petroleum-Gas University of Ploiesti, Ploiesti, Prahova, Romania; 2 Accounting, Banking & Finance Department, Valahia University of Targoviste, Targoviste, Dambovita, Romania; 3 Cybernetics, Economic Informatics, Finance and Accounting Department, Petroleum-Gas University of Ploiesti, Ploiesti, Prahova, Romania; University of Rijeka, CROATIA

## Abstract

Contemporary economies are strongly reliant on energy and analyzing the determining factors that trigger the changes in energy paradigm and their impact upon economic growth is a topical research subject. Our contention is that energy paradigm plays a major role in achieving the sustainable development of contemporary economies. In order to prove this the panel data methodology of research was employed, namely four panel unit root tests (LLC, IPS, F-ADF and F-PP) aiming to reveal the connections and relevance among 17 variables denoting energy influence on economic development. Moreover, it was introduced a specific indicator to express energy consumption per capita. Our findings extend the classical approach of the changes in energy paradigm and their impact upon economic growth and offer a comprehensive analysis which surpasses the practices and policy decisions in the field.

## Introduction

Energy is by far one of the most important vectors in promoting sustainable economic development in contemporary societies. During the years, energy in general, and particularly the energy market has experienced numerous and effective paradigm changes under the pressure of geopolitical and geostrategic threats and supply insecurity. But the diversification of energy supply sources, the promotion of renewables and also the possibility of the enterprises to access the energy exchange market have had a major contribution to the optimization of energy production and consumption. Providing secure energy supply implies the well-functioning of a network of factors including efficiency of energy market and a good intersection of energy production and demand in a fluid and highly competitive context.

Nowadays energy has been transformed into a veritable production factor [1], with a straight influence on the economic growth. The most of literature focus on the relationship between energy and different components of the environmental mechanism–(resource productivity, economic growth, greenhouse emissions related industry, CO2 emissions and complementary components) [2–3], there are new research trends dealing with the relevance of energy in achieving sustainable development and economic growth. In literature [4] has been highlighted a direct and significant influence of energy usage on economic growth on numerous countries surveyed, implicit from the perfective of GDP growth, welfare and resource productivity.

In another study [5] it is point out that economic growth and energy consumption self-influence each other and trigger on short and long run a causal relationship between their components, such as foreign direct investments, relative prices and the financial development of a country [5]. Moreover, the direct link between energy consumption and the income level was also debated by numerous surveys [6–8]. However, the connection between energy and the different components of the economic development is still approached in the literature [9–12] more theoretically rather than analytically.

Following the accelerated development of the production forces in the past century, the post-industrial era brings to attention an unprecedented environmental problem. The last decades have been marked by a series of evolutions in this field whose magnitude, based on the past experience, is expected to be amplified in the coming period. There has to be pointed out that these evolutions are concurrent with the requirements of the continuous economic development as the only known viable source for the unceasing increase of living standards in modern societies and the necessary improvement of the quality of life in traditional ones. In our opinion, the main criterion that should be used to divide the two types of society is represented by the accessible form of energy for the majority of the population.

The traditional societies are characterized by the extensive usage of solid fuels, especially wood, in areas that already experience serious environmental problems, affecting once more the fragile global equilibrium. Not least, some of these countries are denominated as having “emergent economies”, with part of them in the top of the world production of goods, and the environmental effect of their industries is undeniable. As the development is fundamentally based on energy, the access to superior forms of energy (especially electric) for the traditional communities still may be accomplished by means of fossil fuels, confining resources and issuing emissions.

According to the Eurostat data [13], twelve countries in Europe (Slovenia, Croatia, Latvia, Hungary, Lithuania, Slovakia, Poland, the Czech Republic, Romania, Estonia, and Bulgaria) still have an energy intensity over 200 kilograms of oil equivalent (kgoe) per 1000 Euros of GDP.[13]

Analyzing the possibilities of carbon emissions reduction, Caiazza [14] underlines that there are two feasible solutions: the decrease in energy intensity and the improvement of consumption structure. Fundamentally, this development can be achieved by decreasing the share of energy production based on fossil fuels and correlatively increasing the renewable energies, that is, the fuel substitution effect and renewable energy penetration effect.

Many scholars have dealt with the impact of energy patterns on the different components of economic development in contemporary economies. Several examples are worth mentioning, starting with Kraft and Kraft [15] who studied the causal relationship between economic growth and energy consumption within the US economy, continuing with [16] who analyzed the same issue considering six Central American countries between 1980–2004 using a multivariate research framework, or Akinlo [17] considering eleven Sub-Saharan African countries by using the autoregressive distributed lag (ARDL), and Esso and Keho [18] who analyzed the causal link between energy consumption, carbon dioxide (CO2) emissions, and economic growth in the case of 12 selected Sub-Saharan African countries.[18]

Taking into consideration all the above mentioned opinions, studies and differences, we argue that the causal relationship between energy paradigm evolution and economic growth requires an in-depth analysis from a specific perspective. Previous studies and reports have analyzed the correlation between energy consumption and economic growth [19–21] ignoring economic efficiency aspects. In an effort to complete the infield analysis and to properly understand the full economic effects of energy in contemporary economies, by using 17 variables, we aim to extend the current research by investigating the broader economic effects of energy production and taxation [22–23] in promoting sustainable economic development.

The novelty of this paper consists in the design of a specific indicator which expresses the energy consumption per capita and in the use of four panel unit root tests (LLC, IPS, F-ADF and F-PP) to reveal the connections and relevance of considered variables. Moreover, in all these cases the null hypothesis of a unit root is provided against the alternative of stationarity, and all the tests are adapted for the specific requirements of the panel data analysis. Also, the length of the data sets ensures the implementation of the tests and tools throughout the paper to be valid.

## Data series and preliminary results

The latest datasets available on the Eurostat (2016) were employed, which allowed us to include Romania in the study among the other EU-28 countries. In addition, using indicators such as: energy intensity, energy productivity, energy dependence, and resource productivity, allowed the energy performance measuring in the economy. Table 1 presents the variables, the period of data availability, and the symbols employed for each series.

**Table 1:** Description of the variables and data series.

Symbol	Description of the Variable	Time-period availability of the data
***Egrs***	Electricity generated from renewable sources (% of gross electricity consumption)	2004–2014
***Edep***	Energy dependence (%)	1990–2014
***Eint***	Energy intensity (kg of oil equivalent per 1 000 Euro of GDP)	2003–2014
***Epty***	Energy productivity (Euro per kilogram of oil equivalent)	1995–2014
***Gdp***	Real GDP per capita (Euro per inhabitant)	1995–2015
***Gec_a***	Gross inland energy consumption–all products	1990–2014
***Gec_g***	Gross inland energy consumption–gas	1990–2014
***Gec_n***	Gross inland energy consumption–nuclear	1990–2014
***Gec_p***	Gross inland energy consumption–petroleum	1990–2014
***Gec_r***	Gross inland energy consumption–renewable	1990–2014
***Gec_s***	Gross inland energy consumption–solid fuels	1990–2014
***Gec_w***	Gross inland energy consumption–waste	1990–2014
***Pec***	Primary energy consumption (mil. tonnes of oil equivalent)	1990–2014
***Ppre***	Primary production of renewable energy (tonnes of oil equivalent)	2003–2014
***Rpty***	Resource productivity (Euro per kilogram, chain linked volumes– 2010)	2000–2015
***Shrec***	Share of renewable energy in fuel consumption of transport (%)	2004–2014
***Shrft***	Share of renewable energy in gross final energy consumption (%)	2004–2014

Source: authors based on EUROSTAT (2016).

The descriptive statistics of the variables employed in the study is presented in Table 2. As the research is based on the panel data approach, there may be observed that the common period of data availability is 2004–2014. The study takes into consideration major aspects, such as: energy consumption components, energy dependence and energy intensity, as well as energy efficiency, by using energy productivity and resources productivity.

**Table 2:** The descriptive statistics of the employed variables.

	*Egrs*	*Gdp*	*Gec_a*	*Gec_g*	*Gec_n*	*Gec_p*	*Gec_r*	*Gec_s*	*Gec_w*	*Edep*	*Eint*	*Epty*	*Ppre*	*Pec*	*Rpty*	*Shrec*	*Shrft*
** Mean**	22.85	25.42	62.31	14.83	8.43	22.20	5.91	10.55	0.36	37.20	190.82	6.50	5.77	58.43	1.46	16.54	2.85
** Median**	16.10	22.60	28.43	4.41	1.01	9.90	2.78	3.88	0.12	54.30	146.80	6.80	2.51	26.90	1.18	12.40	2.10
** Maximum**	109.60	82.90	351.70	87.75	116.47	123.48	35.41	86.13	4.30	102.50	626.60	14.60	36.02	327.60	3.74	69.20	21.60
** Minimum**	0.00	3.90	0.87	0.00	0.00	0.87	0.00	0.00	0.00	-665.50	68.60	1.60	0.00	0.90	0.24	0.20	0.00
** Std. Dev.**	21.73	16.94	83.17	22.11	21.39	29.71	7.01	17.72	0.65	109.29	100.90	2.75	6.82	78.20	0.90	13.68	2.89
** Skewness**	1.73	1.27	1.92	1.90	4.12	1.78	1.66	2.78	3.89	-5.18	1.55	0.38	1.71	1.91	0.70	1.54	2.21
** Kurtosis**	6.48	4.70	5.76	5.45	20.01	5.04	5.42	10.43	20.84	29.97	5.69	2.62	5.76	5.66	2.59	5.63	12.28
** *J-B* test**	314.2	121.6	291.6	267.4	4660.1	219.5	220.2	1124.4	4938.3	10882.1	220.5	9.6	251.8	283.0	27.6	214.4	1378.1
** Probability**	0.000	0.000	0.000	0.000	0.000	0.000	0.000	0.000	0.000	0.000	0.000	0.000	0.000	0.000	0.000	0.000	0.000
** Sum**	7153.5	7956.4	19502.4	4641	2638	6949	1851	3304	113	11643	59728	2035	1806	18290	458	5177	893
** S.Sq.Dev.**	147361	89582	2157981	152482	142770	275386	15317	97954	132	3726745	3176527	2353	14499	1908075	250	58376	2603
** Obs.**	313	313	313	313	313	313	313	313	313	313	313	313	313	313	313	313	313

Source: authors’ own computations based on Table 2.

## Materials and methodology

The data employed in the current study refers to 29 countries, representing the European Union member states at this moment and Norway. As presented in Table 1, the series are annual, extended over 11 years, running between 2004 and 2014, representing the earliest data available for the *electricity generated from renewable sources*, *share of renewable energy in fuel consumption of transport*, and respectively, *share of renewable energy in gross final energy consumption*. Also, some of the data series start in 2003 (cases of *energy intensity* and *primary production of renewable energy*), or in 2000 (as those regarding *resource productivity*).

It is worth mentioning that the European framework to account for the energy issues, not only renewables, as in the case of *energy intensity* and *resource productivity*, was finally established in 2004. Nevertheless, the present research could benefit from the positive aspects of longer series of data, but, the missing variables named above represents a disadvantage.

In the literature [24], the population represents one of the variables emphasized as a major driving factor for energy consumption and taxation. Intuitively, this approach is correct–the energy consumption is expected to increase both with the number of population and with the prosperity of society, as the access to superior forms of energy represents a key aspect of the modern lifestyle. On the other hand, from the methodological point of view, it is appealing to consider that energy intensity may not represent a fair measure to rank the countries from the energy consumption point of view, as this indicator has two drawbacks:

1. first, it does not consider the population, apparently one of the driving factors in a country’s total energy consumption; and,
2. instead, it takes into account only the GDP, which, usually, tends to decrease with the population and especially with the population growth rate.

Relying on these observations and starting from the available data, we tried to build an indicator in order to express the energy consumption per capita. In this respect, the total consumption of energy per inhabitant (*TE*/*P*) could be considered as a valid measurement in order to account for the population and it was computed using (1):

$$\frac{TE}{P}=\frac{TE}{GDP}\cdot \frac{GDP}{P},$$ (1)

where *TE*/*GDP* accounts for the energy intensity and *GDP*/*P* represents GDP per capita. The results of this computation are presented in Table 3.

**Table 3:** Average energy intensity and energy consumption per inhabitant.

Country	Energy intensity	Total energy consumption per inhabitant
**Denmark**	78.8	3.1
**Ireland**	87.6	3.4
**Norway**	92.2	3.6
**Italy**	109.4	4.3
**Austria**	114.5	4.5
**United Kingdom**	115.3	4.5
**Luxembourg**	117.8	4.6
**Spain**	126.4	5.0
**Germany**	128.1	5.0
**Netherlands**	131.9	5.2
**Greece**	132.3	5.2
**France**	132.8	5.2
**Sweden**	136.0	5.3
**Portugal**	140.9	5.5
**Cyprus**	142.9	5.6
**Malta**	143.2	5.6
**Belgium**	159.9	6.3
**Finland**	191.0	7.5
**Slovenia**	202.4	7.9
**Croatia**	207.6	8.1
**Latvia**	236.3	9.3
**Hungary**	253.6	9.9
**Lithuania**	274.2	10.8
**Slovakia**	279.3	11.0
**Poland**	282.5	11.1
**Czech Republic**	290.4	11.4
**Romania**	298.6	11.7
**Estonia**	377.1	14.8
**Bulgaria**	514.3	20.2

Source: authors based on EUROSTAT (2016).

It may be observed that the classification of the countries according to the total consumption of energy per inhabitant leads to exactly the same results as the classification based on the energy intensity criterion. In our opinion, this result can be equivalent to the fact that energy intensity may express the level of development of a certain country regardless the number of population. Thus, the developed countries succeed in ensuring superior life conditions, concomitant to reduced consumption of energy. Also, for the considered panel of countries, this may represent a valid proof that the number of population does not affect the energy intensity, and, consequently, for the sample period, this variable does not affect the results of the research. This finding is consistent with other similar research results presented in the extant literature [25–27].

Another study [27] with closed conclusions outlines that the main factors that determine the emissions of CO2 are the growth in population and the increase in the general level of income; the evolution of revenues is granted to exert stronger pressures than the growth in population. Interestingly, within the population-effect, the main determinant is not the number but the affluence, whilst the income-effect evidence various evolutions across countries. [27]

Due to increasing interest in the CO2 emissions mitigation, as the global environmental situation tends to worsen, in the literature exists plenty of researches in the interrelated fields of energy production, consumption, taxation, structure, prices, etc., often times in close connection with the economic growth.

The latest evolutions in the analysis of various energy aspects imply using the panel data approach and its subsequent techniques, presented below, due to important econometric advantages: the considerable increase of the sample size, which allows for higher degrees of freedom, more reliable statistical tests, including increasing the power of cointegration ones; the heterogeneity between countries; the reduced collinearity between the considered variables.

In this framework, Granger causality tests represent the main tool in the application of cointegration techniques, in order to observe the error correction mechanism (ECM) and to examine the long and short-run relationships between the considered variables [28]. The underlying idea of employing the causality tests is based on Granger’s [29] proof, according to which when the series are integrated of order one, *I*(1), there might occur linear combinations by virtue of which the series become stationary without differencing.

The series of this type are considered cointegrated and in this context, the existence of a long-run relationship between the integrated variables is a task which is analyzed by using the cointegration analysis. If the cointegration is proved, the issue of differencing, represented by the loss of information on any long-run relationships between variables, is avoided through the ECM, employed to check whether there is any stationary linear combination of non-stationary variables, implying that a long-run equilibrium relationship holds between the variables.

The first step to be taken consists in panel unit root testing. The problem of the unit root is not a key issue in the panel data approach, especially where the length of the time series is relatively reduced, as in the case of the present research. Although testing for the stationarity of panel data is complicated by the inherent heterogeneity of country-section specific parameters, on the other hand, considering cross-sectional units as independent, sometimes, does not represent an appropriate approach.

In order to avoid as much as possible the backward and to best capture the virtuous aspects of employing these tests, we considered using four panel unit root tests, namely LLC [30], IPS[31], and, respectively Fisher-type tests based on ADF [32] and PP tests [33] (herein denoted as F-ADF and F-PP, respectively). All considered tests have, in various forms, the null hypothesis of a unit root (common, in the case of LLC, or for some cross-sections, in the case of the last three) against the alternative of stationarity, and are adapted so as to deal with the requirements specific to the panel data environment. Basically, the autoregressive (AR) model has the form [16] (2):

$$\Delta y_{it}=\rho y_{i,t-1}+\sum_{L=1}^{pi}\theta_{iL}\Delta y_{i,t-L}+\delta X{'}_{it}+\varepsilon_{it},$$ (2)

where, by $i=\bar{1,N}$ are denoted the cross-sections, observed through the time length $t=\bar{1,T}$; *p*_*i*_ represents the lag order, allowed to vary among individuals, and is determined by means of the *t*-statistic of ${\hat{\theta }}_{iL}$ (under the null, (${\hat{\theta }}_{iL}=0$), these *t*-statistics are distributed *N*(0,1), either if *ρ*_*i*_ = 0 or *ρ*_*i*_ < 0); *X*'_*it*_ are the exogenous variables of the model, containing fixed effects and, if necessary, individual trend; by *ρ*_*i*_ are denoted the AR coefficients; *ε*_*it*_ describes a stationary process. If *ρ*_*i*_ < 1, *y*_*i*_ is considered to be low trend-stationary; in case *ρ*_*i*_ = 1, then *y*_*i*_ is not stationary, that is, the series contains a unit root.

Although the LLC test is based upon running distinct augmented Dickey-Fuller (ADF) regressions for each individual, the null hypothesis assumes the existence of a unit root process that is common for all cross sections; that implies a value of *ρ*, the same across sections, against the alternative of no unit root. Among the unit root tests for panel data IPS is the most used; it assume a relaxation in the restriction homogeneity as it is specified for the LLC test, allowing for a coefficient of *y*_*i*,*t*−1_ which can vary among individuals, and putting forward a testing procedure based on the calculation of the $\bar{t}$-statistic as [24]:

$$\bar{t}=\frac{\sum_{i=1}^{N}t_{\rho i}}{N}$$ (3)

where, *t*_*ρi*_ represents the individual *t*-statistic for testing of the null hypothesis in each cross-section of the panel, assumed that every individual contains a unit root: $\rho_{i}=0,\left(\forall \right)i=\bar{1,N}$. The alternative is specified upon [34], [35]:

$$H_{1}:\left\{ \begin{array}{l} \rho_{i}<0,\;\text{for}\;i=\bar{1,N_{1}}\\ \rho_{i}=0,\;\text{for}\;i=\bar{N_{1}+1,N} \end{array}\right.,$$ (4)

namely, it requires that the fraction in the series which are stationary to be nonzero: $\lim_{N\to \infty }\left(\frac{N_{1}}{N}\right)=\gamma ,0<\gamma <1$. In case that the lag order is always zero ($\rho_{i}=0,\left(\forall \right)i=\bar{1,N}$), IPS test stipulates simulated critical values for $\bar{t}$, provided for a different number of individuals *N*, and length in series,

Some researches in the literature [36] points out the underlying idea of such testing, based on the finding that a linear combination of two or more non-stationary series may be stationary. If this type of stationary linear combination exists, the non-stationary time series are said to be cointegrated. The stationary linear combination may be considered as a relationship of long-run equilibrium across the considered variables and it is called cointegrating equation.

In this research, it was opted to assess the cointegration among variables using Kao test for which Gutierrez [37] suggests that it has a higher power than other competing tests, especially in a homogenous panel and that, as in our case, the time series length is relatively short.

The Kao test is based either on a version of the ADF test on the residual (*ε*_*it*_) of the auxiliary regression *ε*_*it*_ = *ρε*_*it*−1_ + *ν*_*it*_, or on the augmented version of the pooled specification [24], [38]:

$$\varepsilon_{it}=\rho \varepsilon_{it-1}+\sum_{j=1}^{p}\lambda_{j}\Delta \varepsilon_{it-j}+\nu_{it}$$ (5)

Under the null hypothesis of no cointegration, the augmented version of the test is constructed as follows [24]:

$$ADF=\frac{t_{\rho }+\frac{\sqrt{6N}{\hat{\sigma }}_{v}}{2{\hat{\sigma }}_{0v}}}{\sqrt{\frac{{\hat{\sigma }}_{0v}^{2}}{{\hat{\sigma }}_{v}^{2}}/+\frac{3{\hat{\sigma }}_{v}^{2}}{10{\hat{\sigma }}_{0v}^{2}}}}∼N\left(0,1\right),$$ (6)

where ${\hat{\sigma }}_{v}^{2}$ is the estimated variance and ${\hat{\sigma }}_{0v}^{2}$ is the estimated long-run variance of the error term. Nevertheless, the Kao test is based on the assumption of a homogenous value of *ρ* across the cross-sections in the panel. Extant literature [39] suggests seven types of residual-based cointegration tests that support this assumption, allowing for considerable heterogeneity: panel *v*-statistic, panel *rho-*statistic, panel *ADF*-statistic, panel *PP*-statistic, group *rho*-statistic, group *ADF*-statistic and group *PP*-statistic.

More specifically, Pedroni [39] tests are based on the estimated residuals of the panel regression, *ε*_*it*_ = *ρ*_*i*_*ε*_*it*−1_ + *ν*_*it*_, under the null of no cointegration, *ρ*_*i*_ = 1. Basically, the panel cointegration test is a test of unit roots in the estimated residuals of the panel. That is equivalent to the expectance of the stationarity of residuals in the case of a cointegrating relation.

Nevertheless, Pedroni [39] suggests that in cases like *rho* and *pp* tests there is a bias to under-reject the null in the case of small samples. The first set (panel tests) comprises four tests, based on pooling and averaging the test statistics for cointegration in the time series across the within-dimension of the panel; in addition, other four values of the same tests are computed as weighted statistics.[39]. The second type (group tests) includes three tests based on pooling the residuals of the regression along the between-dimension of the panel. In the latter case, the limiting distributions are based on limits of piecewise numerator and denominator terms.

For both sets of Pedroni tests, the cointegrating relationship is estimated separately for each cross-section, followed by the pooling of the resulting residuals in order to run the panel tests. The value of the test statistics is then computed as an average of the estimated coefficients corresponding to each cross-section. As a consequence, every test statistics balances the short-run specific dynamics of every member, the fixed individual effects and deterministic trends (within-dimension), as well as the slope coefficients (between-dimension) specific to every cross-section.

In order to evaluate the long-run relationship present between the nonstationary series that proved to be cointegrated from the specific tests presented above, it was used a vector error correction model (VECM) and consequent error correction term. In this situation, a VECM is a restricted autoregression vector (VAR) that may be estimated using a series of different techniques as: dynamics of fully modified OLS (DOLS/FMOLS) models [40], [41] the pooled mean group estimator (PMG) [42] Quasi Maximum Likelihood (QML), or Generalized Method of Moments (GMM).

This type of model involves the estimation of the long-run bi-variate relationship with the inclusion of a lead and lags of the differenced explanatory variable, providing better results than other competing techniques. The VEC has the cointegration relations embedded into the specification; as a result, it allows the long-run behavior of the endogenous variables only towards converge to their cointegrating relationships, whilst allowing for short-run adjustment dynamics. The cointegration term is denominated as error correction term (ECT), as the deviation from the long-term equilibrium is gradually corrected through a series of short-run partial adjustments. The GMM [39], [34] represents a suitable technique in the estimation of a panel VARs, using lags of the endogenous variables as instruments for computing unbiased and consistent estimates.

The model can be specified as follows (7):

$$\begin{array}{l} \Delta E\;{\mathrm{int}}_{i,t}=\alpha_{i}^{e\mathrm{int}}+\beta_{i}^{e\mathrm{int}}ECT_{i,t-1}^{e\mathrm{int}}+\sum_{j=1}^{m}\delta_{ij}^{e\mathrm{int}}\Delta E\;{\mathrm{int}}_{i,t-j}+\sum_{s=1}^{q}\gamma_{1,is}^{e\mathrm{int}}\Delta Gec\_r_{t-s}+\ldots +\sum_{s=1}^{v}\gamma_{9,is}^{e\mathrm{int}}\Delta Shrec_{t-v}+u_{it}\\ \ldots \\ \Delta Shrec_{i,s}=\alpha_{i}^{shrec}+\beta^{v}ECT_{i,t-1}^{shrec}+\sum_{j=1}^{m}\delta_{ij}^{shrec}\Delta E\;{\mathrm{int}}_{i,t-j}+\sum_{s=1}^{q}\gamma_{1,is}^{shrect}\Delta Gec\_r_{t-s}+\ldots +\sum_{s=1}^{v}\gamma_{9,is}^{shrec}\Delta Shrec_{t-v}+w_{it} \end{array}$$ (7)

where $ECT_{i,t-1}^{e\mathrm{int},\ldots ,shrec}$ are the lagged residuals, derived from the long-run cointegrating relationship; $\delta_{ij}^{e\mathrm{int},\ldots ,shrec}$ and $\gamma_{1,..,9}^{e\mathrm{int},\ldots ,shrec}$ represent the coefficients of short-run adjustment, whereas *u*_*it*_ and *w*_*it*_ represent the disturbance terms, assumed to be uncorrelated and have zero mean.

The specification of these models must exhibit an instrumental variable estimator, due to the correlation between the lagged endogenous variables and the error term. The inclusion of fixed effects into the model is in line with Arellano and Bover’s [34] approach, aimed at removing the unobserved heterogeneity, whereas the use of orthogonal deviations, which are similar to differences from the mean approach, removes the unobserved heterogeneity in the panel members.

According to Granger et al [28], the long-term causality is evaluated via the significance of the *beta* coefficients (that is, the coefficients of *ECT*) using the standard *t*-statistic, whereas the short-run causality is measured through the joint-significance of the lagged explanatory variables. The long-run causality examination by the significance of the *beta* coefficients is based on their determinant role within the long-run relationship in the cointegrating process.

Their values express the speed in elimination of the deviations from the long-run equilibrium, through changes in each variable; therefore, the process movement on the way described by the significance of the *ECT* coefficients is taken for granted as a permanent one. Bond (2002) considers that the Arellano-Bover [43] approach may have some advantages over other approaches to dynamic panel models. In addition, the *ECT* coefficient, that represents the speed of adjustment following an exogenous shock, is expected to be negative in order to ensure the stability of the model.

The choice of the panel VECM approach in this research relies on its flexibility, allowing the use of heterogeneous panels and correction for both heteroskedasticity standard errors and serial correlation. Methodologically, there has to be pointed out that, in the case of no consistent evidence of cointegration, as the applicability of ECM is limited for the situations when the series are *I*(1), the ECT are not included in the EC models, and the standard Granger causality models are estimated without an ECT. Also, in cases of no cointegration, the inclusion of all the considered variables in the ECM has to be based on their stationarity. However, the literature suggests that, if added anyway, they report insignificant results [24].

## Results and discussions

According to the literature review [1–2], [5], there are many recent contributions addressing various connections of the energy sector. Most of these researches are in the field of the economic growth and energy taxation [22], trying to assess the limits of energy taxation as a factor positively correlated with development, and, more specifically, to sustainable development, as the mankind aggression towards the planetary environment represents an incontestable reality. The first studies in this field were conducted from a local perspective, focused on a comparison between the results of VAR models for various countries.

Nevertheless [42] in the first studies launched in the panel data approach, the observations sets used to account for a small number of countries, sometimes divided into groups, considering their geography, economic development, or other criteria; that led to homogenous characteristics of respective groups. To put it different, one of the key aspects of using the panel data approach is that they allow for heterogeneity control. It is known that European Union countries are homogeneous with regard to policy settings whereas there are important disparities when considering their development level. Nevertheless, [44] represents one of the most comprehensive studies that employed the multiple facets of the panel data approach.

Our panel consists of 29 cross-sections, represented by the European Union member countries at this moment, plus Norway. There are also other countries that adhered to the Eurostat report framework, but the data availability concerning them is usually limited to fewer than five years. However, these data refer to the candidate countries, which, in this respect are following the specific roadmap in order to ensure the improvement of their energy balance as a condition for joining the EU.

We have chosen to use the Eurostat data due to its compatibility across all the countries in the panel, which, from the econometric point of view, further ensures the compatibility between the variables across the considered countries. The results from the running of the panel unit root tests are presented in Table 4.

**Table 4:** Panel unit root tests.

Variable	LLC		IPS		ADF-Fisher Chi-square		PP-Fisher Chi-square	
	Level	Differenced	Level	Differenced	Level	Differenced	Level	Differenced
***Egrs***	11.12	-6.93***	12.95	-2.495***	7.178	100.98***	12.304	142.747***
***Gdppc***	-3.98***	-9.067***	-1.02	-3.49***	66.46	96.18***	117.99***	107.87***
***Gec_a***	1.025	-20.46***	3.65	-10.835***	31.34	226.266***	43.14	359.156***
***Gec_g***	1.779	-14.688***	3.592	-7.58***	21.474	165.981***	25.338	226.121***
***Gec_n***	-3.56***	-7.418***	-0.78	-4.744***	38.782	84.051***	66.424***	178.853***
***Gec_p***	-0.729	-15.059***	2.886	-7.648***	29.802	172.51***	32.188	243.737***
***Gec_r***	-0.288	-14.951***	4.161	-7.581***	29.535	170.325***	54.537	244.763***
***Gec_s***	-3.13***	-17.03***	0.873	-9.1***	50.625	19.752***	63.736	276.284***
***Gec_w***	-1.305*	-9.639***	2.254	-4.347***	37.565	110.863***	42.588	178.677***
***Ed***	-1.541*	-18.785***	2.021	-10.910***	35.163	226.376***	66.946	355.114***
***Ei***	-3.57***	-14.254***	1.816	-6.972***	39.532	159.11***	66.756	242.913***
***Epy***	-1.266	-14.399***	3.299	-7.608***	32.732	169.646***	36.798	256.776***
***Ppre***	0.636	-15.141***	4.97	-7.691***	22.551	172.056***	38.929	253.275***
***Pec***	1.277	-20.691***	3.241	-11.892***	38.431	242.894***	48.411	352.796***
***Rpy***	-1.998**	-13.542***	1.905	-6.913***	36.884	157.994***	46.796	213.458***
***Shrec***	3.497	-11.015***	7.722	-5.493***	7.867	139.173***	12.284	193.040***
***Shrft***	0.414	-10.758***	3.356	-5.228***	35.491	131.696***	53.293	165.121***

Notes: Lag length determined upon the modified Hannan-Quinn Criteria. Probabilities for the LLC and IPS tests are computed assuming asymptotic normality. Probabilities for Fisher tests are computed using an asymptotic Chi-square distribution. All tests equations include individual constant term („fixed effects”). *Differenced* refers to series resulted from first-difference.

***, **, * indicates the significance at 1, 5, and 10% levels (one tailed test).

After checking for stationarity, the main finding is that, generally, the cross-sections and the cross-sections units are cointegrated of order one. Only the using of the LLC test reported a significant part (though, a minority) of the cross-sections to be stationary, for various levels of significance. Nevertheless, previous research [34], [45] pointed out that the results using of the IPS test are reasonably satisfactory and generally better than those obtained from running of the LLC test [45]. As a consequence, we take for granted the result that the series are cointegrated of order one, *I*(1). The results of using the IPS tests are also largely confirmed by the results issued through employing the F-ADF and F-PP tests.

The significant results of the pairwise Granger causality tests for the considered dataset are presented in Table 5; as in [28], [35] we test for cointegration in both directions, with both variables acting as the dependent variable.

**Table 5:** The significant results of the pairwise Granger causality tests.

Variables	Egrs	Gdp	Gec_a	Gec_g	Gec_n	Gec_p	Gec_r	Gec_s	Gec_w	Edep	Eint	Epty	Ppre	Pec	Rpty	Shrec	Shrft
***Egrs***	-						15.9^***^				3.2^**^	3.19^**^	14.67^***^				
***Gdppc***		-									4.32^**^	11.3^***^					
***Gec_a***			-								4.79^***^	5.5^***^	28.4^***^	4.23^**^	4.28^**^		2.43*
***Gec_g***				-	4.66^**^		28.0^***^	12.88^***^				2.53*	23.4^***^	8.43^***^	4.54^**^		
***Gec_n***				7.29^***^	-	6.02^***^	7.63^***^		11.48^***^				7.42^***^	5.14^***^			
***Gec_p***				8.64^***^	16.81^***^	-	32.97^***^		15.79^***^			2.74*	27.28^***^		5.87^***^		
***Gec_r***	5.9^***^				2.56*		-			4.57^**^			3.26^**^				9.63^***^
***Gec_s***	2.41*			6.30^***^	13.37^***^	10.23^***^	19.12^***^	-	8.60^***^		3.16^**^		18.62^***^	5.11^***^			
***Gec_w***					14.52^***^	12.06^***^	10.70^***^		-				15.24^***^				
***Edep***	14.6^***^					3.41^**^	7.46^***^			-		16.98^***^	7.04^***^		4.38^**^		3.66^**^
***Eint***	5.57^***^										-						
***Epty***	13.19^***^						6.35^***^			2.36*		-	5.64^***^				
***Ppre***	4.54^**^				3.05^**^		4.63^**^			3.99^**^			-				9.77^***^
***Pec***	3.15^**^			13.9^***^	10.85^***^	12.15^***^	33.22^***^	6.54^***^	4.03^**^		4.46^**^	5.03^***^	27.85^***^	-	3.99^**^		
***Rpty***		3.57^**^				2.86*								2.57*	-		
***Shrec***							19.33^***^					8.27^***^	17.86^***^		2.85*	-	
***Shrft***				3.11^**^			5.29^***^				9.50^***^		4.51^**^				-

Notes: In the first columns, the explanatory variable in the cointegrating relation; the headings, the dependent variable.

***, **, * Indicates rejection of the null hypothesis of no cointegration at the at the 1, 5, and 10% levels of significance.

As we have already mentioned before, the extant literature and the statistical measurements (such as energy intensity) exhibit a bias for linking the GDP with the energy consumption. Despite these approaches, the results for the considered countries and in the outlined period express a decoupling of the two indicators.

These results suggest that variables *eint*, *giec_r*, *giec_p*, *edep*, *pec*, *ppre*, *rpty*, *epty egrs*, *shrec*, may be found in a contegration relationship. As a consequence, we used the Pedroni tests, adapted to the specific Eviews software package, which supports at most seven cointegrated series: as a result, the series were split up into three subgroups, in order to be suitable to run a Pedroni test, as follows: the first subgroup, containing the series: *egrs*, *gec_p*, *gec_r*, *edep*, *eint*, *epty*, *ppre;* the second subgroup, consisting of the series *pec*, *rpty*, *shrec*, *edep*, *eint*, *ppre*, *gec_r*; and the third made up of the series: *pec*, *rpty*, *shrec*, *egrs*, *gec_p*, *gec_r*, and *epty*. This *triangular* approach was intended to ensure a double check for all the considered series. The results of Pedroni cointegration tests are presented in Table 6.

**Table 6:** Results of Pedroni and Kao panel cointegration tests.

Test statistic	Subgroup 1		Subgroup 2		Subgroup 3	
	Statistic	Weighted-stat	Statistic	Weighted-stat	Statistic	Weighted-stat
**Panel *v*-Statistic**	-2.757	-3.931	-2.399	-4.280	-3.816	-4.502
**Panel *rho*-Statistic**	6.497	6.567	5.236	6.117	5.961	5.918
**Panel *PP*-Statistic**	-5.359^***^	-8.146^***^	-9.536^***^	-13.299^***^	-16.460^***^	-19.828^***^
**Panel *ADF*-Statistic**	-1.759^**^	-2.686^***^	-5.987^***^	-4.876^***^	-6.288^***^	-7.065^***^
**Group *rho*-Statistic**	9.076	-	8.223	-	8.091	-
**Group *PP*-Statistic**	-16.115^***^	-	-18.679^***^	-	-27.281^***^	-
**Group *ADF*-Statistic**	-2.377^***^	-	-6.632^***^	-	-9.520^***^	-
**Kao test**	*ADF t*-Stat		-4.082^***^	*ρ* = -0.304 (-6.812)^***^		

Notes: Lag length determined upon the modified Schwartz Info Criterion. All tests equations include individual constant term (“fixed effects”). For the coefficient *ρ* afferent to the Kao test *t-Stat* value in parenthesis.

***, **, * indicates the rejection of the null hypothesis of no cointegration at the 1, 5, and 10% levels of significance (one-tailed test).

Analyzing Table 6, one may observe mixed results giving varying conclusions, but in the majority of cases the null hypothesis of no cointegration is rejected. Even when considering Pedroni’s suggestion, according to which in cases like *rho* and *pp* tests there is a bias to under-reject the null in the case of small samples (see the previous paragraph), it is possible to observe that, in all considered cases for the *rho* test the null is rejected, whilst, for the *pp* test the results are opposed. Moreover, taking into account the result of the Kao test, as well as the results of the pairwise Granger causality tests, we consider the inclusion of the ECT in the VECM. Table 7 presents the results from the GMM estimation of the VECM using the Arellano-Bover approach.

**Table 7:** Estimation of ECT in the Vector Error-Correction Model.

Variables	ECT coefficients (*t*-statistic)	Speed of adjustment (*t*-statistic)	Lag coefficient (*F*-statistic)
***EINT***	1.000	0.000162(0.833)	-1.151*
***GEC_R***	-5.315(-8.235)***	-0.0114(-1.274)	10.042***
***GEC_P***	-2.659(-20.593)***	0.215(17.964)***	29.894***
***EDEP***	-0.0056 (-2.721)***	0.67(3.688)**	5.275***
***PEC***	-0.596(-11.607)***	0.412 (15.948)***	41.810***
***PPRE***	2.988 (4.835)***	-0.0072 (-0.766)	9.704***
***RPTY***	-0.655(-2.285)***	-0.0054(-2.752)***	1.649*
***EPTY***	-0.179(2.176)**	-0.0176(-3.562)***	4.724**
***EGRS***	0.057(3.311)***	-0.085(-3.673)**	3.322**
***SHREC***	-0.117(-4.175)***	-0.0439(-2.467)***	0.407

Notes: Lag length: 1, 2.

***, **, * indicates the significance at the 1, 5, and 10% levels of significance.

The significant results in the estimated ECT highlight various situations of the considered variables, as follows: values generally tending towards zero, which indicates that the long-term adjustment process is slow, as in the case of variables *edep*, *egrs*, *shrec*, and *epty*; in cases of *primary energy consumption* and *resource productivity* one can observed that the system has the tendency to overcome an important part of the disequilibrium in each period; in addition, there is a tendency to over-shoot the long-term equilibrium, namely in cases of *gross inland energy consumption*, for both considered types, petroleum and renewable energy; on the other hand, the positive values indicate the variables whose evolution disturbs the system from the long-run equilibrium, such as the *primary production of renewable energy* and, with a lower impact, the *energy generated from renewable sources*.

Regarding the short-run causal effect, the results indicate important evidence in favor of all the considered variables except for *shrec*, as evidenced by the significance of the lagged explanatory variables. The exception represents the share of renewable energy in total energy consumption, situation that can be explained by the reduced values within the series. As regards the speed of adjustment, the results presented in Table 7 provide evidence in favor of the significance of almost every considered variable within the process, except for *eint*, *gec_r*, and *ppre*. This situation can be explained also by the reduced share of renewable energies in total consumption, in most of the countries under analysis. The speed of adjustment for *gec_p*, *edep*, and *pec* is significantly and positively signaled, whereas they are negatively signaled in cases such as *ppre*, *rpty*, *epty*, *egrs*, and *shrec*.

## Conclusions

As the environmental initiatives gain momentum, there can be observed a reverse movement of the developing countries that outrun the leading countries that relied in their development on using the fossil resources and now, due to their policy in favor of the global diminution of emissions, they prevent the developing countries from accessing their own resources and sentence them to perpetual underdevelopment. In this seemingly contradictory evolution, the production and consumption of energy, compliant with environmental requirements, represent the only viable key in order to ensure the sustainable development of society and economy. However, the issue does not regard only the countries mainly characterized by traditional societies.

The paper demonstrates that the increasing trends for energy demands in contemporary economies are obvious and it is necessary to improve both the energy paradigm and the measurements in order to optimize the efficiency. Most researches [46–50] in the field underline the relationship between the economic growth and energy as a positive factor correlated with sustainable development. We contend now that some change is necessary in the energy production and consumption paradigm, both at the European Union level and inland level, in order to achieve economic performance and become more environmentally friendly. It was argued that the factors that disturb the equilibrium of the system in the long run are the primary production of renewable energy and, with a lower magnitude, the energy generated by renewable sources.

The findings of this study should be considered in the light of its limitations, for instance the fact that our panel consists of only 29 cross-sections, represented by the European Union member countries at this moment, and Norway. We contend that it will be easier to start a debate regarding the role of energy paradigm changes on economic growth while there is a capacity to understand the evolution of technological gains and innovation promotion in society. Finally, future research could address the uniqueness of each complementary aspect of energy economics by interpolating more variables into an extended analysis and comparing the results with previous research studies.
